# Monitoring PD-L1 positive circulating tumor cells in non-small cell lung cancer patients treated with the PD-1 inhibitor Nivolumab

**DOI:** 10.1038/srep31726

**Published:** 2016-08-24

**Authors:** Chiara Nicolazzo, Cristina Raimondi, MariaLaura Mancini, Salvatore Caponnetto, Angela Gradilone, Orietta Gandini, Maria Mastromartino, Gabriella del Bene, Alessandra Prete, Flavia Longo, Enrico Cortesi, Paola Gazzaniga

**Affiliations:** 1 Department of Molecular Medicine, Circulating tumor cells Unit, Sapienza University of Rome; 2 Department of Radiological, Oncological and Pathological Sciences, Division of Medical Oncology, Sapienza University of Rome.

## Abstract

Controversial results on the predictive value of programmed death ligand 1 (PD-L1) status in lung tumor tissue for response to immune checkpoint inhibitors do not allow for any conclusive consideration. Liquid biopsy might allow real-time sampling of patients for PD-L1 through the course of the disease. Twenty-four stage IV NSCLC patients included in the Expanded Access Program with Nivolumab were enrolled. Circulating tumor cells (CTCs) were analyzed by CellSearch with anti-human B7-H1/PD-L1 PE-conjugated antibody. PD-L1 expressing CTCs were assessed at baseline, at 3 and 6 months after starting therapy, and correlated with outcome. At baseline and at 3 months of treatment, the presence of CTCs and the expression of PD-L1 on their surface were found associated to poor patients outcome. Nevertheless, the high frequency of PD-L1 expressing CTCs hampered to discriminate the role of PD-L1 in defining prognosis. Conversely although CTCs were found in all patients 6 months after treatment, at this time patients could be dichotomized into two groups based PD-L1 expression on CTCs. Patients with PD-L1 negative CTCs all obtained a clinical benefit, while patients with PD-L1 (+) CTCs all experienced progressive disease. This suggests that the persistence of PD-L1(+) CTCs might mirror a mechanism of therapy escape.

The process of adaptive immune resistance was first described to explain how cancer cells evade an otherwise effective immune response, through the expression of molecules that actively turn off cytotoxic tumor-specific T-cells^1^. Inhibitory immune checkpoints play a crucial role in the maintenance of immune homeostasis, mitigating autoimmunity. Among those, PD-1/PD-L1 axis recently got considerable attention in the context of anticancer immunotherapy^2^. The interaction between PD-1 (programmed cell death protein 1) and its ligand (PD-L1) is involved in the peripheral effector phase of T-cell activation and results in peripheral immunologic tolerance. The strong rationale for the immune checkpoint inhibition as anticancer therapy paved the way for a wide number of studies conducted to investigate the efficacy of this therapeutic approach in different cancers. Following the Phase III CheckMate-017 trial that evaluated the PD-1 inhibitor Nivolumab in metastatic non-small cell lung cancer (NSCLC) after prior platinum-based chemotherapy, the U.S. Food and Drug Administration (FDA) has fast-tracked the approval of Nivolumab to extend its use to patients with previously treated metastatic NSCLC, regardless of PD-L1 expression^3^. This latter point is due to an evident biological issue limiting the reliability of PD-L1 expression in tumor samples as predictive biomarker of response to Nivolumab. Although PD-L1 can be detected by immunohistochemistry (IHC) on tumor or immune cells, its expression is controversial in predicting which patient might benefit from therapy^4^. In that respect, it is notable that the majority of patients with PD-L1 positive tumor do not respond to PD-1 pathway blockade, suggesting that PD-L1 expression might not be necessary for achieving objective response. To date, the low positive predictive value of PD-L1 test in cancer biopsy makes it an unacceptable biomarker to drive treatment selection^5^. Furthermore, the up-regulation of PD-L1 is a dynamic biomarker and cannot be adequately represented by a static snapshot, as is the case with tumor tissue biopsy sample. The observation that PD-L1 status is a dynamic parameter together with the lack of standardization in available assays hamper its use as ideal predictive biomarker in tumor biopsy due to both technical and biological issues, being its expression extremely variable according to the time and site of biopsy^6^. Liquid biopsy, through the accessible and repeatable isolation of tumor cells into the bloodstream^7^, might by contrast allow for a dynamic characterization of PD-L1 expression which can be monitored through the course of the disease. Since circulating tumor cells (CTCs) arise from tumor cells, it is conceivable that, under evolutionary pressure, they might share some of the immune escape mechanisms inherent to tumor cells. In this view, retaining PD-L1 might represent one of the mechanisms that CTCs use to survive immune system/immunotherapy attack. Aims of the present study were 1) to investigate PD-L1 expression in CTCs isolated from patients with NSCLC treated with the PD-L1 inhibitor Nivolumab 2) to monitor any change in PD-L1(+) CTCs during the course of treatment and 3) to clarify whether PD-L1(+) CTCs might represent a predictive biomarker to anti-PD-1 directed therapies.

## Results

### Patient characteristics

Characteristics of the 24 metastatic NSCLC patients enrolled in the study are summarized in Table 1. CTCs status was assessed before initiation of therapy (baseline) and at 3 and 6 months after the beginning of treatment (at the time of the first two radiological reassessment of disease status). A total of 24, 15 and 10 patients had a blood draw at baseline, 3 months and 6 months after initiation of therapy, respectively. Indeed, between the baseline and the 3 months blood draw six patients died and three withdrew from the study (blood sample inadequate for CellSearch analysis), while after the first follow-up blood draw three patients died and two withdrew from the study (one drop out of therapy for drug toxicity, one blood sample inadequate for CellSearch analysis). CTC status was correlated to the outcome of patients 6 months after starting treatment (Table 2).

### CTCs enumeration and PD-L1 status in CTCs at baseline

CTC status at baseline was positive in 20/24 (83%) and negative in 4/24 (17%) patients. The number of CTCs detected ranged from 1 to 20 (median number of CTCs 5.2). As negative controls, we tested blood samples from 15 healthy controls and confirmed that none of the samples were positive for CTCs (Data not shown).

Among CTC (+) patients at baseline, 14 (70%) experienced disease progression or died, while 5 (25%) had a disease control (defined as stable disease SD or partial response PR) at the second follow up time (6 months after starting treatment). In one patient (5%) the 6 months follow up was not available, since he dropped out from the study for drug toxicity.

**Figure 1:**
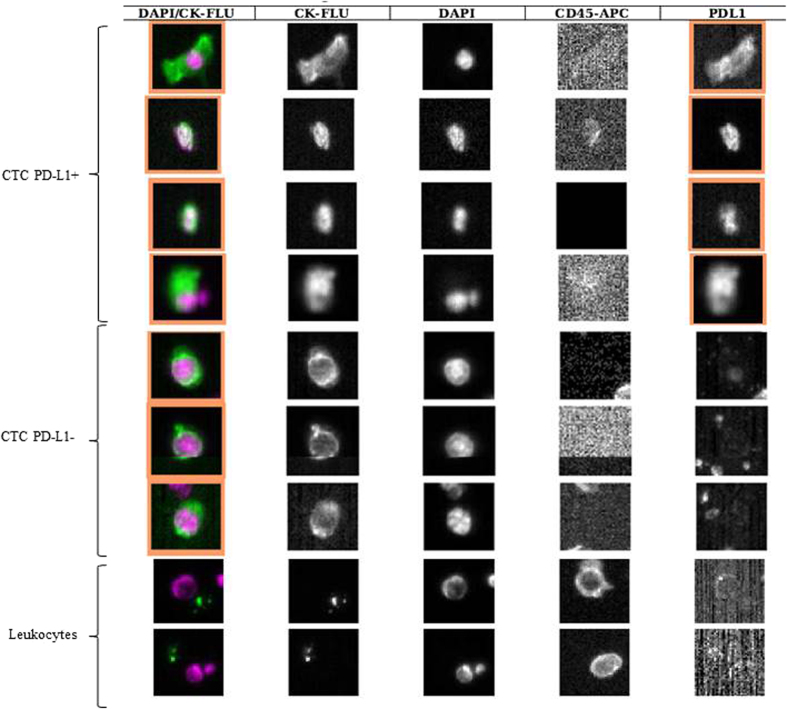
CellSearch analysis of circulating tumor cells isolated from one patient with NSCLC treated with Nivolumab (pt. 7 of Table 2). The patient had 7 CTCs, of which 4 PD-L1(+). Figure shows the elongated shape of PD-L1(+) CTCs compared to PD-L1 negative.

PD-L1 status in CTC at baseline was evaluable in the group of 20 patients with detectable CTC at baseline. Among these 20 patients, 19 (95%) showed a subpopulation of PD-L1 expressing CTCs. The presence of PD-L1 positive CTCs was found independent of EGFR mutational status and history of smoking. The number of PD-L1(+) CTCs varied from 1 to 10 (median number 3.6 cells) and the fraction of PD-L1(+) CTCs ranged from 25 to 100% of the whole number of detectable CTCs. The intensity of PD-L1 staining varied between patients and within the same blood sample. The vast majority of PD-L1(+) CTCs presented an irregular shape, mostly elongated and with peripheral nucleus, as compared to the more frequent round shape observed in PD-L1(−) CTCs (Fig. 1). Of the 19 patients with PD-L1(+) CTCs at baseline, 5 (26%) achieved a clinical benefit and 14 (74%) had a progressive disease after 6 months of treatment. At baseline, only one patient had CTCs not expressing PD-L1 and withdrew from the study after 3 months of treatment, due to drug toxicity.

### CTCs enumeration and PD-L1 status in CTCs at 3 months of treatment

A total of 15 patients had a second blood draw for CTCs enumeration, after 3 months from initiation of therapy. Among them,10 (67%) had detectable CTCs, of whom 7 (70%) experienced a progression of disease and 3 (30%) a clinical benefit (2 SD, 1PR) at 6 months of follow up. Changes in levels of CTCs between baseline and 9 to14 weeks after initiation of therapy was frequently observed. Particularly, an increase in CTCs number was found in 6 patients (1 SD, 5 PD), a decrease (or drop to zero) was observed in 7 patients (3 PD, 2 RP, 1 SD, 1 dropped out) and 2 patients were found persistently negative for CTCs presence (both SD).

PD-L1 status in CTCs at the first radiological restaging was evaluated in the group of 10 patients with detectable CTCs. Among CTCs-positive patients, all had PD-L1 expressing CTCs. The number of PD-L1(+) CTCs varied from 1 to 53 (median number 6.4 cells) and the fraction of PD-L1(+) CTCs ranged from 25 to 100% of the whole number of detectable CTCs. At the first re-evaluation of PD-L1 status in CTCs, an increase of PD-L1(+) CTCs compared to baseline was observed in 6 patients (5 PD, 1SD), while a decrease in PD-L1(+) CTCs was observed in 4 patients (1 SD, 2 PR, 1 PD). Both the proportion of PD-L1 expressing CTCs among the whole CTCs population and the correlation between PD-L1 status in CTCs at the first re-evaluation and the radiological restaging of disease at 6 months were not dissimilar compared to the results obtained at the baseline analysis.

### CTCs enumeration and PD-L1 status in CTCs at 6 months of treatment

**Figure 2:**
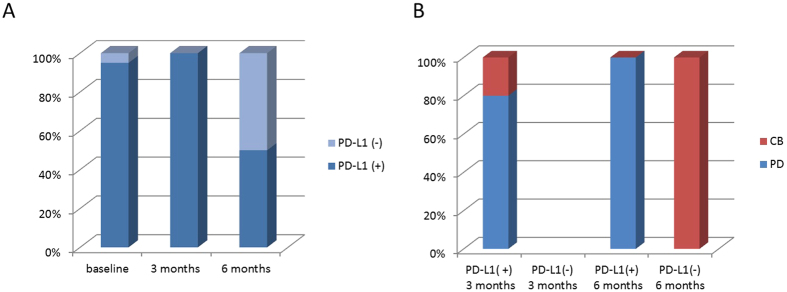
PD-L1 status in CTCs at baseline, at the first follow-up (3 months of treatment) and at the second follow-up (6 months of treament). A high proportion of CTCs expressed PD-L1 at baseline and at the first re-evaluation, while the percentage of PD-L1(+) CTCs significantly dropped at the second re-evaluation (**A**). Correlation between PD-L1 status in CTCs and outcome of patients at 3 and 6 months of treatment. At 3 months all CTCs detected were found PD-L1(+). At 6 months, patients could be dichotomized into two groups based on whether or not CTCs expressed PD-L1. Interestingly, patients with PD-L1(–) CTCs all obtained a clinical benefit, while patients with PD-L1(+) CTCs all experienced progression of disease (**B**). CB:clinical benefit, PD:progression of disease.

A total of 10 patients had a third blood draw at 6 months from initiation of treatment. All had detectable CTCs. Of these, 5 had a PD. 4 had a SD and 1 a PR. An increase in CTCs number compared to the 3 months blood draw was found in 6 patients (3 PD, 2 SD and 1 PR), a decrease (or drop to zero) was observed in 3 patients (2 PD, 1SD) and 1 patient was found persistently positive for CTCs presence (SD). Among patients with detectable CTCs at the second radiological re-evaluation, 5/10 (50%) had PD-L1(+) CTCs. All these patients experienced a progression of disease. Interestingly, at 6 months of treatment we observed a population of PD-L1(−) CTCs in 50% of patients, who all achieved a clinical benefit (4 SD and 1 PR) (Fig. 2). An increase in PD-L1(+) CTCs compared to the 3 months blood draw was found in 3 patients (all PD), a decrease or drop to 0 in 3 patients (2 SD, 1 PR). Two patients were found persistently positive for PD-L1(+) CTCs (all PD), and the remaining 2 were found persistently negative for PD-L1(+) CTCs (both SD).

## Discussion

To date, the controversial results on the predictive value of PD-L1 expression in NSCLC and response to PD-1/PD-L1 inhibitors do not allow for any conclusive consideration regarding the static assessment of PD-L1 as a biomarker^8^. The feasibility of PD-L1 assessment in CTCs has been previously described in breast cancer^9^. We for the first time monitored PD-L1 expressing CTCs in patients with stage IV NSCLC progressing post prior systemic treatment, in the first 6 months of treatment with Nivolumab. At baseline and at 3 months of treatment two observations were particularly intriguing. The first was that the vast majority of patients had detectable CTCs at the baseline blood draw, in contrast with the previously reported low CTC detection rate in NSCLC patients at the CellSearch analysis, which does not exceed 40% in the international literature^10^. The high rate of CTCs detection is probably due to the fact that all the patients analysed were included in the Expanded Access Program, being thus affected by heavily pretreated (second-line and beyond) metastatic NSCLC. The second observation was that at both baseline and at 3 months after starting treatment almost all CTCs expressed PD-L1, irrespective of mutational status of EGFR, of the tumor histology and history of smoking. Thus, although both CTC presence and PD-L1 expression were found consistently associated to poor outcome of patients, the extremely high frequency of PD-L1 expressing CTCs at baseline and at the first re-evaluation hampered to effectively discriminate the role of CTCs themselves compared to that of PD-L1 in defining prognosis. In other words, at baseline as well as at 3 months after starting treatment, the lack of a population of CTCs negative for PD-L1 expression did not allow to address the real prognostic and predictive significance of PD-L1 in CTCs. Conversely although CTCs were still found in all patients 6 months after treatment, at this time point patients could be dichotomized into two groups based on whether or not CTCs expressed PD-L1. Interestingly, at 6 months of treatment, patients with PD-L1 negative CTCs all obtained a clinical benefit, while patients with PD-L1(+) CTCs all experienced progression of disease. This observation suggests that PD-L1 expression on CTCs starts assuming a more clear predictive significance late in course of treatment. This is consistent with data obtained in Checkmate 017 trial, comparing Nivolumab vs docetaxel in pretreated advanced NSCLC patients, where the two overall survival curves show a delayed separation, which starts approximately at 6 months^3^. This slow response to checkpoint inhibitors is explained with the time necessary to reactivate immune system^11^. It is thus conceivable that 3 months of treatment are not sufficient to allow immune system to eliminate PD-L1(+) CTCs, so that a liquid biopsy at this time point might be not useful to discriminate patients candidate to respond. Conversely, the persistence of PD-L1 (+)CTCs at 6 months might mirror a mechanism of therapy escape. We further observed that most persistent PD-L1 positive CTCs displayed an unusual elongated spindle-like morphology with an enlarged tail composed of the nucleus, compared to PD-L1 negative CTCs, which were mostly small and rounded. Interestingly, these morphological abnormality of CTCs have been correlated to poor prognosis in different tumor types^12^. It has been suggested that these elongated CTCs may represent a small population of partial EMT-transformed cancer cells^13^, which can be still captured by CellSearch, due to the fact that they might retain some epithelial features (cytokeratins, EpCAM), as a consequence of a partial transformation process. A striking association has been reported between PD-L1 expression and induction of epithelial-mesenchymal transition (EMT) in different cancer types, supporting a potential mechanism for EMT associated immunosuppression^14^. In the troubled search for a biomarker, all studies have focused on PD-L1 expression in primary tumors as a candidate biomarker of response^15^. Data from literature suggest that, although the overall response rate (ORR) to Nivolumab in NSCLC is higher in patients with PD-L1 positive tumors compared to PD-L1 negative, still most PD-L1 positive tumors do not respond to anti-PD-1 directed immunotherapy, nor does lack of PD-L1 expression exclude the possibility of response^16^,^17^. According to a recent analysis^18^, the ORR to Nivolumab in a group of 511 NSCLC patients with PD-L1 positive tumors ranges from 16% to 50%, confirming that PD-L1 in tumor tissues represents a very imperfect biomarker. This imperfection might reflect the lack of standardization in available assays, the confusion about the cut-off values, the variable expression of PD-L1 according to the time and site of biopsy, and the wide expression of PD-L1 observed in cancer cells and in other cell types constituting the cancer microenvironment. The use of a liquid biopsy, able to reveal the dynamic nature of resistance mechanisms in solid tumors, might solve some of these questions. We wondered why in this subset of patients PD-L1 positive CTCs looks like a marker of immunotherapy resistance. In lung cancer, evidence has been provided that PD-L1 overexpression is correlated with poor survival of patients^19^. Intratumoral CD8^+^ T cell exhaustion, which largely contributes to the failure of cancer elimination by immune system, was found to be mediated primarily by tumor cell PD-L1 expression^20^. Since PD-L1 positive cancer cells are often found at the tumor invasion front^21^, the hypothesis that cells with more aggressive features and ready to migrate into bloodstream might retain PD-L1 as a shield to evade immune response in a foreign microenvironment sounds appealing. This is the first study planned to investigate the association of PD-L1(+) CTCs with response to anti PD-1 checkpoint immunotherapy. We speculate that the persistence of PD-L1 expressing CTCs into bloodstream during the course of treatment might mirror a mechanism of immunotherapy escape adopted by cancer cells. Whether PD-L1 is retained from the primary tumor or acquired after detachment as a result of selective pressure needs to be further clarified through the comparative analysis of primary tumors and liquid biopsies. The major limitations of this observational study are the small population size which precluded the feasibility of a statistical analysis, and the lack of information concerning PD-L1 status in the primary tumors. This last point is due to the expanded access program, in which Nivolumab was approved irrespective of PD-L1 status on primary tumor. Specifically, the assessment of PD-L1 and TILs status on tumor biopsies would have been useful to clarify whether patients who did not respond to Nivolumab were characterized by PD-L1 positive expression and TIL deficiency, which would have been suggestive for a constitutively dominant expression of PD-L1 and intrinsic immune resistance^22^. A comparison of PD-L1 status in different microenvironments, specifically primary tumor and blood, which is mandatory to this purpose, is currently under investigation in a population of patients candidate to first line treatment with Nivolumab, in which the tumor tissue analysis is feasible. Finally, in the patient population described in the present study, a longer follow up time might allow to clarify the significance of PD-L1 trend in CTCs from patients with durable response.

## Materials and Methods

### Patients

Twenty-four (24) patients with stage IV NSCLC progressing post prior systemic treatment, included in an Expanded access program with Nivolumab were enrolled. Eligible patients were 18 years of age or older, with an Eastern Cooperative Oncology Group (ECOG) performance-status score of 0 or 1. Patient demographic and baseline characteristics are shown in Table 1.

### Blood sample collection

7.5 mL of whole blood were collected into CellSave Preservative tubes (Janssen) containing EDTA and a cell fixative from patients and healthy normal volunteer donors, prior informed consent. Samples were maintained at room temperature and processed within 96 h. The methods were carried out in accordance with relevant guidelines. The study protocol was approved by the local ethical Committee of the Policlinico Umberto I, Sapienza University of Rome (Italy), protocol n. 668/09. Informed consent was obtained from all patients.

### CTC detection

Each blood sample was processed and analyzed by CellSearch System (Janssen). For all experiments we used CellSearch CXC Kit that contains a ferrofluid-based capture reagent and immunofluorescent staining reagents. The ferrofluid reagent consists of nanoparticles with a magnetic core surrounded by a polymeric layer coated with anti-Epithelial Cell Adhesion Molecule (EpCAM) antibody for capturing CTCs. After immunomagnetic capture and enrichment, the following fluorescent staining reagents are added for identification and enumeration: anti-CK-Fluorescein Isothiocyanate (FITC), 4′-6-Diamidino-2-phenylindole (DAPI) and anti-CD45-Allophycocyanin (APC). CellSearch CXC Kit leaves a channel open for assessment of an additional marker on CTCs. We have added anti-human B7-H1/PD-L1 Phycoerythrin (PE)-conjugated antibody (R&D System) into user-defined marker channel. An event was classified as CTC when it exhibits the phenotype EpCAM+, CK+, DAPI+, CD45- and positive or negative for PD-L1.

### Cell line and culture conditions

H441 (ATCC) human lung cancer cell line was selected based on the PD-L1 expression previously described^8^. Cell line was cultured in RPMI 1640 medium, supplemented with 10% fetal bovine serum (FBS) and 1% penicillin-streptomycin (EuroClone SPA) at 37 °C in 5% CO_2_ and 95% air.

### Western blot

To characterize H441 cell line as appropriate positive control, a Western blot analysis was performed. Cells were washed twice in cold PBS, collected and, after centrifugation, solubilized in 10 mMTris-HCl, 160 mMNaCl, 1 mM EGTA, 1% deoxycholic acid, 1% Triton, 0.1% SDS and a complete mini-protease inhibitor cocktail tablet (SIGMA Chemicals Company). The cells were left to lyse one ice for 30 minutes and, after centrifugation at 12000 g for 30 minutes, the supernatant was then collected. Protein concentration was determined by bicinchoninic acid (BCA) assay. Protein extract (30 μg) was incubated at 100 °C for 10 minutes and separated on 4–12% SDS-PAGE gel, blotted onto PVDF membrane (GE Healthcare) and probed overnight at 4 °C with following primary antibodies: mouse monoclonal anti-pan-cytokeratins (2A4), mouse monoclonal anti-PD-L1 (1B12), mouse monoclonal anti-IgG1 (isotype control) (NCG01) (Abcam) and mouse monoclonal anti-EpCAM (C-10) (Santa Cruz Biotechnology). Membranes were washed three times for 10 minutes in TBS and 0.1% Tween-20 and left to incubate with the appropriate secondary antibodies for 1 h at room temperature. After three washes, immunoreactive bands were visualized by enhanced chemoluminescence (PerkinElmer). Mouse anti-actin (Santa Cruz Biotechnology) served as a protein loading control.

### Optimization of parameters

**Figure 3:**
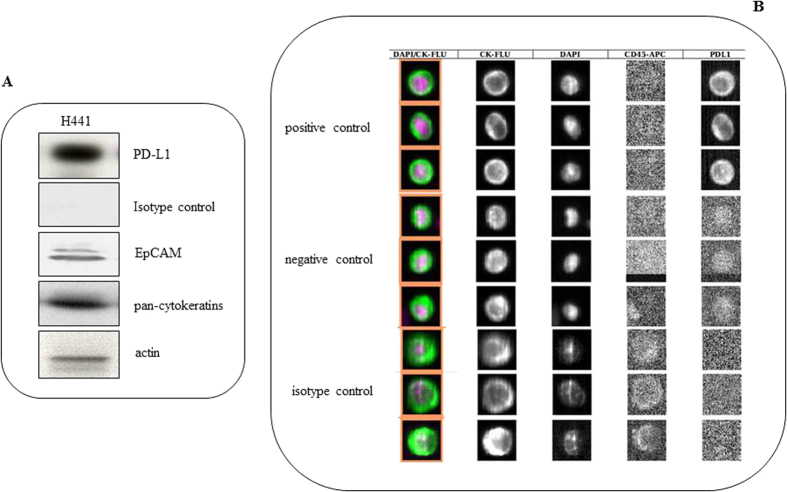
Panel A: Western Blot of EpCam, pan-cytokeratins, PD-L1 and isotype control performed in H441 cell line used for spiking experiments. Panel B: Optimization of parameters for evaluation of PD-L1 positive CTCs by CellSearch in control cell line spiked into healthy donor blood. PD-L1 was detected in 4^th^ channel only in the control cell line, and absent in isotype control, to confirm the specificity of binding between primary antibody and its target. A weak background signal was found in the negative control.

To optimize the parameters for user-defined marker, a series of spiking experiments was performed. To this purpose, defined numbers (700) of H441 cell were spiked into 7.5 mL of blood samples from healthy volunteer in triplicate for CellSearch analysis. The final antibody concentration (0.06 μg/mL) was determined as that producing an optimal staining. Briefly, for one sample, 1 μL of antibody stock solution (25 μg/mL) was diluted in PBS and CXC dilution buffer for a final volume of 450 μL. The diluted antibody was then added to CellSearch reagent cup and placed in the position one of the carrier reagent tray of the CellTracks Autoprep system. The MagNest cartridges were scanned using CellSearch Analyzer II at optimal exposure time (2.5) for PD-L1 visualization in user-defined marker channel within 24 h. We processed H441 cell line in absence of marker (negative control) under the same analysis parameters to account for background signal that may be associated with the exposure time being used to analyze samples. The isotype control, anti-mouse IgG1 PE-conjugated antibody (R&D System), was used at the same concentration to confirm the specificity of binding between primary antibody and its target (Fig. 3).

## Additional Information

**How to cite this article**: Nicolazzo, C. *et al*. Monitoring PD-L1 positive circulating tumor cells in non-small cell lung cancer patients treated with the PD-1 inhibitor Nivolumab. *Sci. Rep.* **6**, 31726; doi: 10.1038/srep31726 (2016).

## Figures and Tables

**Table 1:** Patients characteristics.

AGE	
Median	61
Range	52–79
**GENDER**	
Male	18 (75%)
Female	6 (25%)
**ECOG PS**	
0	12 (50%)
1	12 (50%)
**LINE OF THERAPY**	
2th	18 (75%)
3th	6 (25%)
**SMOKING STATUS**	
Current or former smoker	22 (92%)
Never smoked	2 (8%)
**TUMOR TYPE**	
adenocarcinoma	17 (70%)
squamous cell carcinoma	7 (30%)
**EGFR MUTATION**	
yes	2 (8%)
**ALK mutation**	
positive	1 (4%)

**Table 2:** Total CTCs and PD-L1(+) CTCs number at baseline, at 3 and 6 months of treatment.

Pt	Baseline		3 months			6 months		
	CTC (n.)	PD-L1(+) CTC	CTC (n.)	PD-L1(+) CTC		CTC (n.)	PD-L1(+) CTC	
1	4	1	2	1	PD	1	1	†
2	9	4	n.a	n.a	†	n.a	n.a	
3	17	8	n.a	n.a	†	n.a	n.a	
4	2	1	1	1	†	n.a	n.a	
5	20	8	2	2	PR	2	0	SD
6	0	0	0	0	SD	2	0	SD
7	7	4	78	53	†	n.a	n.a	
8	9	9	n.a	n.a	†	n.a	n.a	
9	1	1	5	5	PD	3	0	SD
10	0	0	0	0	PR	4	0	SD
11	0	0	1	1	PD	3	3	PD
12	3	3	n.a	n.a	†	n.a	n.a	
13	1	1	5	4	†	n.a	n.a	
14	0	0	1	1	PD	3	3	†
15	10	10	1	1	PR	3	0	PR
16	3	1	5	2	PD	3	2	PD
17	4	1	n.a	n.a	†	n.a	n.a	
18	1	0	0	0	PR	n.a	n.a	/
19	2	2	0	0	SD	2	2	PD
20	2	1	0	0	PD	n.a	n.a	PR
21	5	4	n.a	n.a	†	n.a	n.a	
22	3	3	n.a	n.a	SD	n.a	n.a	SD
23	3	3	n.a	n.a	PD	n.a	n.a	PD
24	1	1	n.a	n.a	SD	n.a	n.a	PD

n.a (not applicable): unavailability of blood sample (due to death, inadequacy of blood sampling or drop out) † death; PD: progression of disease; SD: stable disease; PR: Partial response
